# Mechanisms of antibody-dependent enhancement and its research progress in animal coronaviruses

**DOI:** 10.1186/s13567-026-01837-4

**Published:** 2026-09-16

**Authors:** Zhiding Zhou, Yueyue Liu, Meilin Yang, Cunyi Qiu, Jing Xia, Yong Huang, Yefei Zhou

**Affiliations:** 1 College of Veterinary Medicine, Sichuan Agricultural University, Huimin Road, Wenjiang, Chengdu, 611130 Sichuan China; 2 Department of Food Science, Nanjing Xiaozhuang University, Nanjing, 211171 China; 3 Gansu Polytechnic College of Animal Husbandry & Engineering, Wuwei, 733006 China

**Keywords:** Antibody-Dependent Enhancement (ADE), classification, mechanisms, animal coronaviruses, SARS-CoV-2

## Abstract

Animal coronaviruses are associated with serious diseases in livestock and poultry, resulting in substantial economic losses worldwide. Antibody-dependent enhancement (ADE) is a phenomenon whereby pre‑existing pathogen specific antibodies may paradoxically exacerbate viral infections. In this review, we synthesize current classifications of ADE mechanisms, presenting a unified framework comprising two major categories subdivided into five distinct mechanistic subsets. Given the limited research on ADE in animal coronaviruses, we use SARS‑CoV‑2 as a case study to explore five potential ADE mechanisms associated with coronavirus infection. This review aims to provide a valuable reference for elucidating ADE mechanisms in animal coronaviruses.

## Introduction

Despite significant advances in understanding antibody-dependent enhancement (ADE), no universally accepted classification framework for its underlying mechanisms has been established. ADE has been historically classified into extrinsic ADE (antibody-mediated enhancement of viral entry) and intrinsic ADE (suppression of innate antiviral responses by internalized immune complexes) [1–4]. An alternative mediator‑based system categorizes ADE into Fc receptor‑mediated, complement receptor‑mediated, and antibody‑induced conformational changes [5]. Despite these distinctions, a commonality emerges: all these mechanisms ultimately enhance target cell infection. Accordingly, we propose to unify ADE mechanisms that result in increased pathogen burden under the term "antibody‑dependent increased pathogen load" (ADIPL). Recently, Wells et al. [6] proposed an outcome-based framework comprising ADIPL, antibody-dependent serum resistance (ADSR), antibody-dependent enhancement of inflammation (ADEI), and anti-cytokine autoantibodies (ACAs). In this review, we adapt this framework for viral infections by: (i) incorporating complement receptor-mediated ADE and antibody-induced conformational changes into ADIPL; (ii) excluding ADSR, which has no viral counterpart; and (iii) expanding antibody-dependent immune evasion to include ACA-mediated direct IFN neutralization. To clearly illustrate how our proposed classification scheme relates to and differs from the existing systems described above, we have summarized these comparisons in Table 1. In this adapted framework, ADIPL refers to antibody-mediated processes that increase viral burden, including Fc receptor- or complement receptor-mediated enhancement of infection [5], antibody-induced conformational changes that promote membrane fusion and internalization [7], and antibody-dependent immune evasion through suppression of antiviral gene expression. These mechanisms are typically associated with sub-neutralizing concentrations or low-affinity antibodies and correspond to the conventional concept of “classical ADE,” in which pre-existing antibodies enhance viral entry, replication, or overall pathogen load.

**Table 1:** **Comparison of previous ADE classification systems with the unified framework proposed in this review**

Existing classification basis	Existing terminology	Underlying mechanism/feature	Proposed unified term (This Review)	Rationale for unification	Reference
Kinetic stage of infection	Extrinsic ADE	Enhanced viral entry into target cells, expanding cellular tropism	ADIPL	Both extrinsic and intrinsic pathways ultimately lead to an increased viral load (pathogen burden) in the host	[8]
	Intrinsic ADE	Suppression of intracellular innate immune signaling (e.g., type I IFN), enhancing viral replication per cell			[2]
Molecular mediators	Antibody-Dependent Immune Evasion	Antibody-dependent suppression of antiviral gene expression; presence of certain autoantibodies in peripheral blood that can directly neutralize IFNs	ADIPL	All these mediator-specific pathways converge on enhanced viral internalization or replication, resulting in a higher pathogen load	[9, 10]
	FcγR-mediated ADE	Viral entry via activating/inhibitory Fc receptors			[11]
	Complement receptor (C3b/C1q)-mediated ADE	Viral entry via complement receptors or enhanced attachment via C1q			[12, 13]
	Antibody-Dependent Conformational Changes in Viral Proteins	Antibody binding triggers spike protein rearrangements, enhancing membrane fusion			[5, 7]
Pathophysiological outcome	Often described as "immunopathology" or "VAERD" in specific contexts	Antibody-antigen complexes drive excessive FcγR-PRR crosstalk, triggering cytokine storms and tissue damage, without necessarily increasing viral load	ADEI	This represents a distinct pathogenic outcome that is not characterized by increased pathogen burden but by exacerbated inflammation and immunopathology, warranting a separate classification	[6, 14–16]

However, ADE does not invariably manifest as increased pathogen load; antibodies can also exacerbate disease severity during infection by amplifying pathological inflammation. Specifically, antibody-pathogen complexes can trigger FcγR-PRR (pattern recognition receptor) crosstalk, leading to amplified inflammatory responses—a mechanism designated "ADEI" [6]. When patients produce non-neutralizing or sub-neutralizing antibodies that bind viral antigens without blocking or clearing infection, excessive Fc-mediated effector functions or immune complex formation can drive enhanced inflammation and immunopathology [17]. Previous studies suggest that the most plausible ADE mechanism associated with COVID-19 pathology involves antibody-antigen immune complex formation mediating excessive activation of immune cascades within lung tissue [17, 18].

Given the well-characterized ADE mechanisms in human coronaviruses such as SARS-CoV-2, it is of considerable scientific and veterinary importance to systematically review the current state of knowledge regarding ADE in animal coronaviruses. The subfamily *Orthocoronavirinae* comprises four genera: *Alphacoronavirus* (including TGEV and PEDV, which infect piglet intestine), *Betacoronavirus* (including SARS-CoV-1, MERS-CoV, and SARS-CoV-2, which infect human respiratory tract), *Gammacoronavirus* (including IBV in chickens), and *Deltacoronavirus* (including PDCoV in piglets) [19]. Given their pathogenic capacity, broad host spectrum, and cross‑species transmission potential, coronaviruses remain a major research focus. However, studies on ADE mechanisms in animal coronaviruses are severely lacking and largely remain at the phenotypic level. This review therefore aims to reclassify ADE mechanisms and, using SARS‑CoV‑2 as a case study, provide a valuable reference for elucidating ADE in animal coronaviruses.

## Classification and underlying mechanisms of ADE

ADE during viral infection can be broadly classified into two distinct categories: ADIPL and ADEI. ADIPL is typically mediated through enhanced cell internalization via Fc receptors, complement receptors, or conformationally altered viral proteins, or via antibody-mediated suppression of the antiviral effects of innate immune effector molecules, leading to an increased viral burden. This form of ADE has been extensively studied in classic examples of antibody-enhanced viral infections, including Dengue virus (DENV) [20–22], Human immunodeficiency virus (HIV) [23], and Ebola virus (EBOV) [24]. For many years, ADIPL was considered the predominant manifestation of ADE. However, during the COVID-19 pandemic, multiple clinical reports revealed that administration of the SARS-CoV-2 monoclonal antibody cocktail casirivimab/imdevimab could exacerbate pathological inflammation during infection, by triggering cytokine storms and more severe lung injury [25, 26]. These observations brought ADEI into sharper focus. ADEI is typically driven by excessive Fc-mediated effector functions or immune complex formation, and its clinical phenotype is characterized by increased disease severity and elevated inflammatory markers [27]. This type of ADE has also been observed in patients infected with other viruses, including Measles virus (MV) and Respiratory syncytial virus (RSV) [26].

### ADIPL

#### Fc receptor-mediated ADE

**Figure 1:**
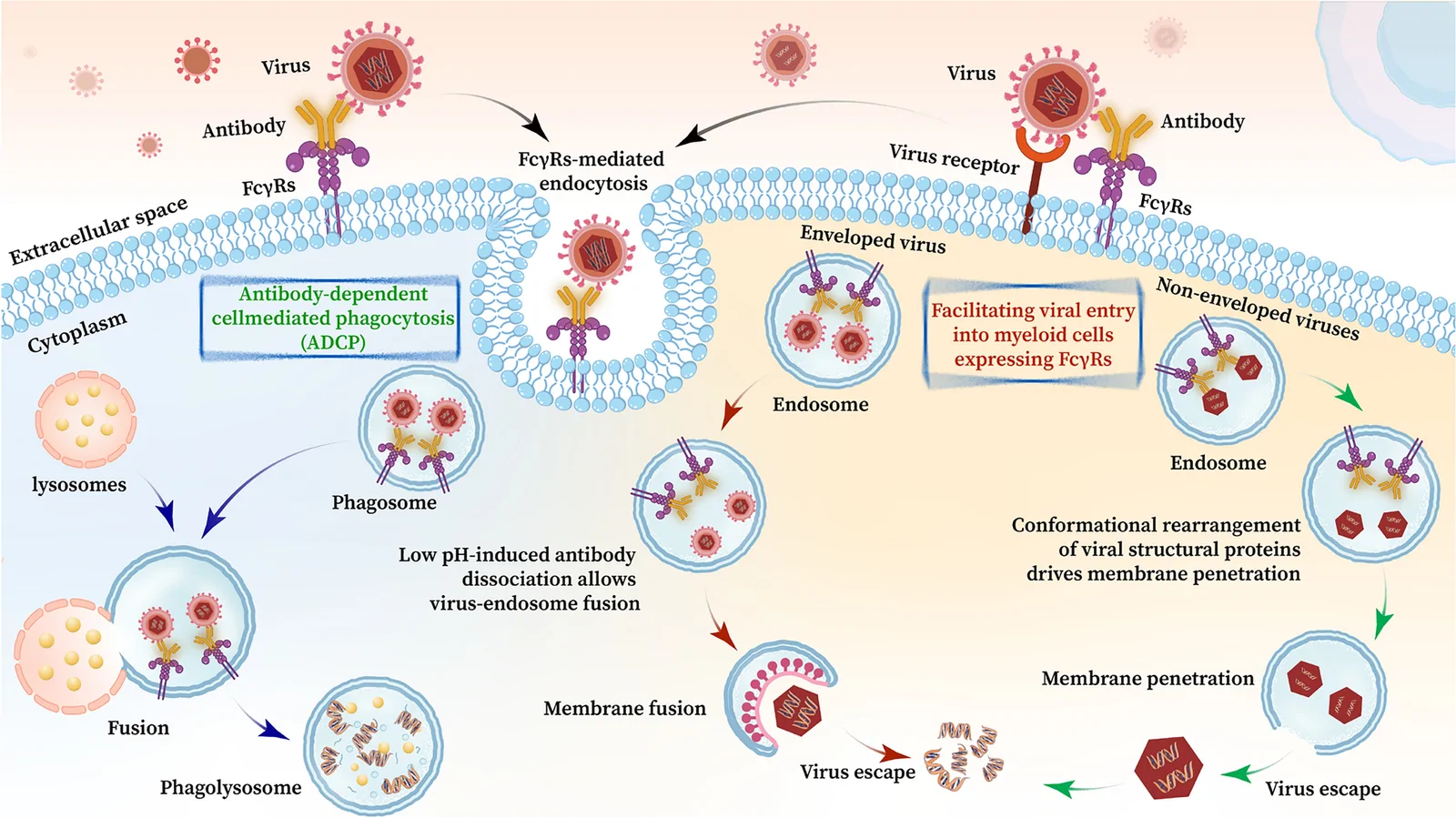
**Fc receptor-mediated ADE.** Antibody-opsonized virions bind FcγRs and are internalized through endocytic or phagocytic pathways. Under non-enhancing conditions, internalized particles are delivered to lysosomes for degradation and antigen presentation. During ADE, some viruses avoid rapid degradation, escape from endosomal compartments, or exploit FcγR-associated signaling, thereby promoting productive infection or immune modulation.

Fcγ receptors (FcγRs) are key receptors for the antibody Fc region, categorized into activating or inhibitory types, and are widely expressed on the surface of macrophages, monocytes, and neutrophils. The activating Fcγ receptors include FcγRI, FcγRIIa, FcγRIIc, and FcγRIIIa. While, FcγRIIb is the sole inhibitory Fc receptor; it is a glycosylphosphatidylinositol (GPI)-anchored protein incapable of signal transduction [28]. This FcγR‑mediated signaling cascade triggers phagocytosis, culminating in phagosome formation and subsequent fusion with lysosomes, where the acidic environment and hydrolytic enzymes degrade the viral particle [29–31]. Viral antigens are processed and presented to CD4+ T cells via MHC class II, initiating adaptive immunity, while macrophages secrete cytokines that recruit additional immune cells. This process is known as antibody‑dependent cellular phagocytosis (ADCP) [32]. Under normal physiological conditions, cargo internalized via FcγRs -mediated phagocytosis is delivered to lysosomes for degradation. However, viruses that exploit ADE have evolved sophisticated strategies to subvert this destructive pathway (Figure 1). For example, when the immune complex formed by dengue virus (an enveloped virus) and the enhancing antibody 2C8 is internalized, exposure to the acidic environment of the endosome reduces the affinity of the antibody for the virus, thereby releasing the virus from the complex and allowing it to fuse with the endosomal membrane, which results in the direct release of the viral genome into the cytoplasm [33]. Conversely, non-enveloped viruses have evolved distinct mechanisms involving membrane-modifying proteins that perforate the endosomal membrane, facilitating the escape of their genome into the cytoplasm [8, 34, 35]. Furthermore, it has been shown that DENV can engage the leukocyte immunoglobulin-like receptor B1 (LILRB1) concurrently with FcγR. This co-ligation recruits the SH2 domain-containing phosphatase−1 (SHP-1), which attenuates FcγR-mediated ADCP and diverts DENV-containing phagosomes to less acidic compartments, thereby preventing rapid lysosomal degradation [36]. Through these evasion strategies, viruses effectively circumvent or delay their destruction within the lysosomal pathway. Consequently, Fc receptor-mediated ADE can enable viral entry into cells that lack the canonical virus receptor, such as certain myeloid cells, epithelial cells, and endothelial cells, thereby broadening viral tropism. An additional mechanism, termed FcR-virus receptor interdependent ADE, involves an interaction between the antibody-bound FcγR and the viral receptor itself. For example, it has been demonstrated that specific neutralizing antibodies against MERS-CoV, upon binding the viral receptor-binding domain (RBD), can also engage cell surface Fc receptors [7]. In this context, the antibody-Fc receptor complex effectively mimics the function of the authentic coronavirus receptor, dipeptidyl peptidase−4 (DPP4), thereby mediating MERS-CoV entry and exacerbating infection.

#### Complement receptor-mediated ADE

**Figure 2:**
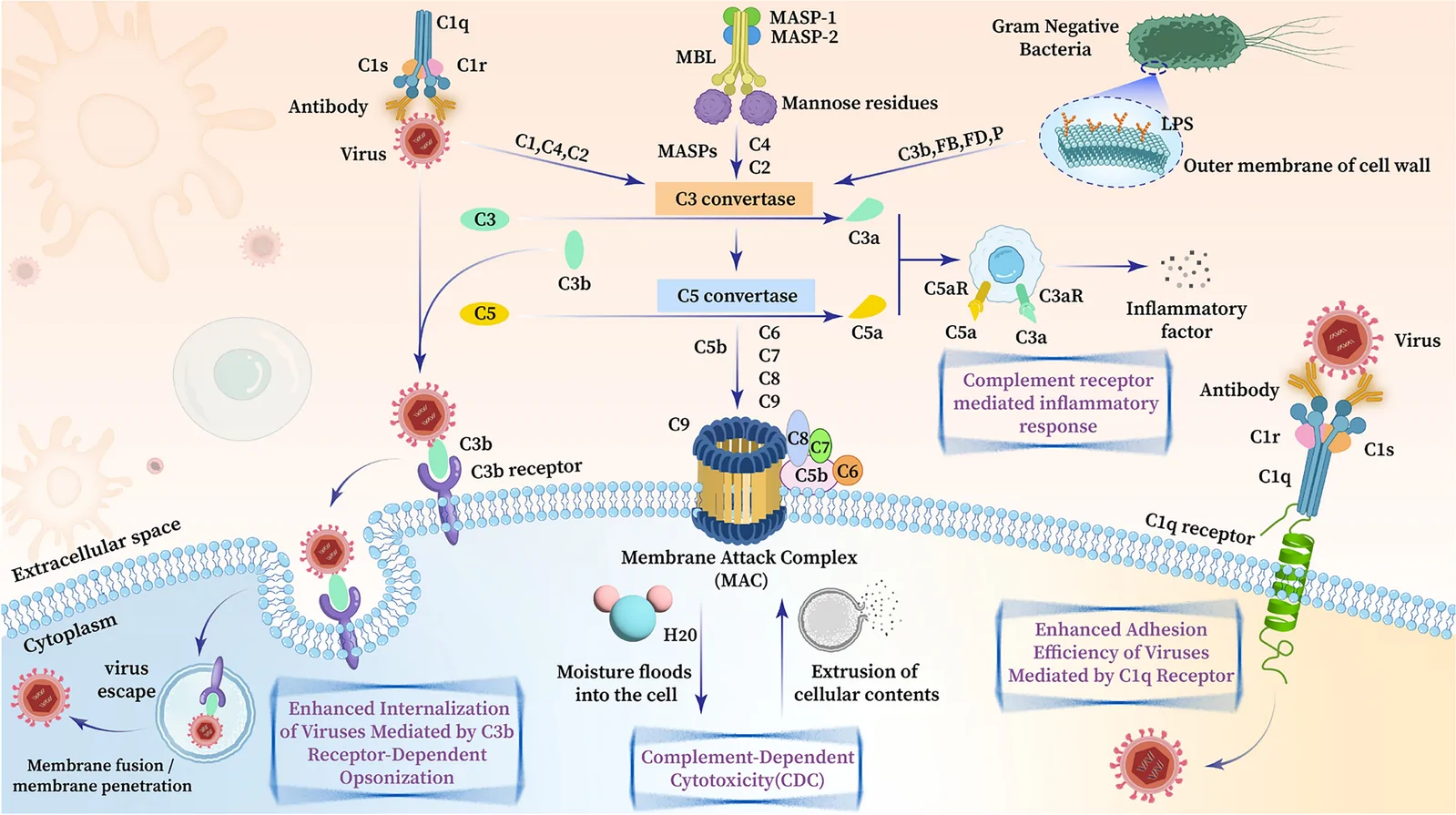
**Complement receptor-mediated ADE.** Antibody-virus complexes may recruit complement components and become decorated with C3 fragments, allowing interaction with complement receptors and enhanced uptake. Alternatively, C1q may bridge immune complexes to C1q receptors and increase viral attachment independently of downstream complement activation.

The classical complement pathway is initiated when C1q binds to antibody Fc regions within immune complexes, activating a proteolytic cascade that generates C3 convertase (C4b2a) and cleaves C3 into C3a (anaphylatoxin) and C3b (opsonin) [37]. The classical, lectin, and alternative pathways converge at C3 cleavage, leading to three downstream outcomes: (i) inflammation via C3a and C5a; (ii) cell lysis via membrane attack complex (MAC) formation; and (iii) opsonization, in which C3b and its degradation products (iC3b, C3dg) engage complement receptors (CR1, CR3, CR4, CR2) on target cells, triggering phagocytic signaling and mediating complement receptor-dependent ADE (Figure 2) [38–41]. For cells expressing CR3 (CD11b/CD18) or CR4 (CD11c/CD18), such as monocytes and macrophages, iC3b deposited on antigen–antibody complexes can bind to these receptors, mediating viral internalization [42, 43]. For cells expressing CR2, such as B lymphocytes, C3dg on immune complexes binds with high affinity to CR2 [44, 45]. This interaction can lead to internalization of the entire complex into B cells, where the virus may replicate or be disseminated via B cell trafficking, potentially contributing to B cell dysfunction and immunosuppression [46]. Beyond receptor-mediated effects involving C3 fragments, C1q itself can directly bind to virus-antibody complexes and enhance infection. For instance, it has been demonstrated that anti-primate erythroparvovirus 1 (B19V) antibodies in human serum can enhance B19V entry into endothelial cells via the surface protein CD93, which serves as a cellular receptor for C1q. Notably, this direct enhancing role of C1q does not require the participation of downstream complement factors [12]. Another study revealed that in the presence of enhancing antibodies, EBOV-antibody-C1q complexes bind to C1q receptors on the cell surface, leading to enhanced viral entry. Importantly, this increased infectivity was not attributable to more efficient internalization into endosomes, but rather to enhanced attachment of EBOV particles to the surface of human kidney 293 cells when both enhancing antibodies and C1q were present [47].

#### Antibody-dependent conformational changes in viral proteins

**Figure 3:**
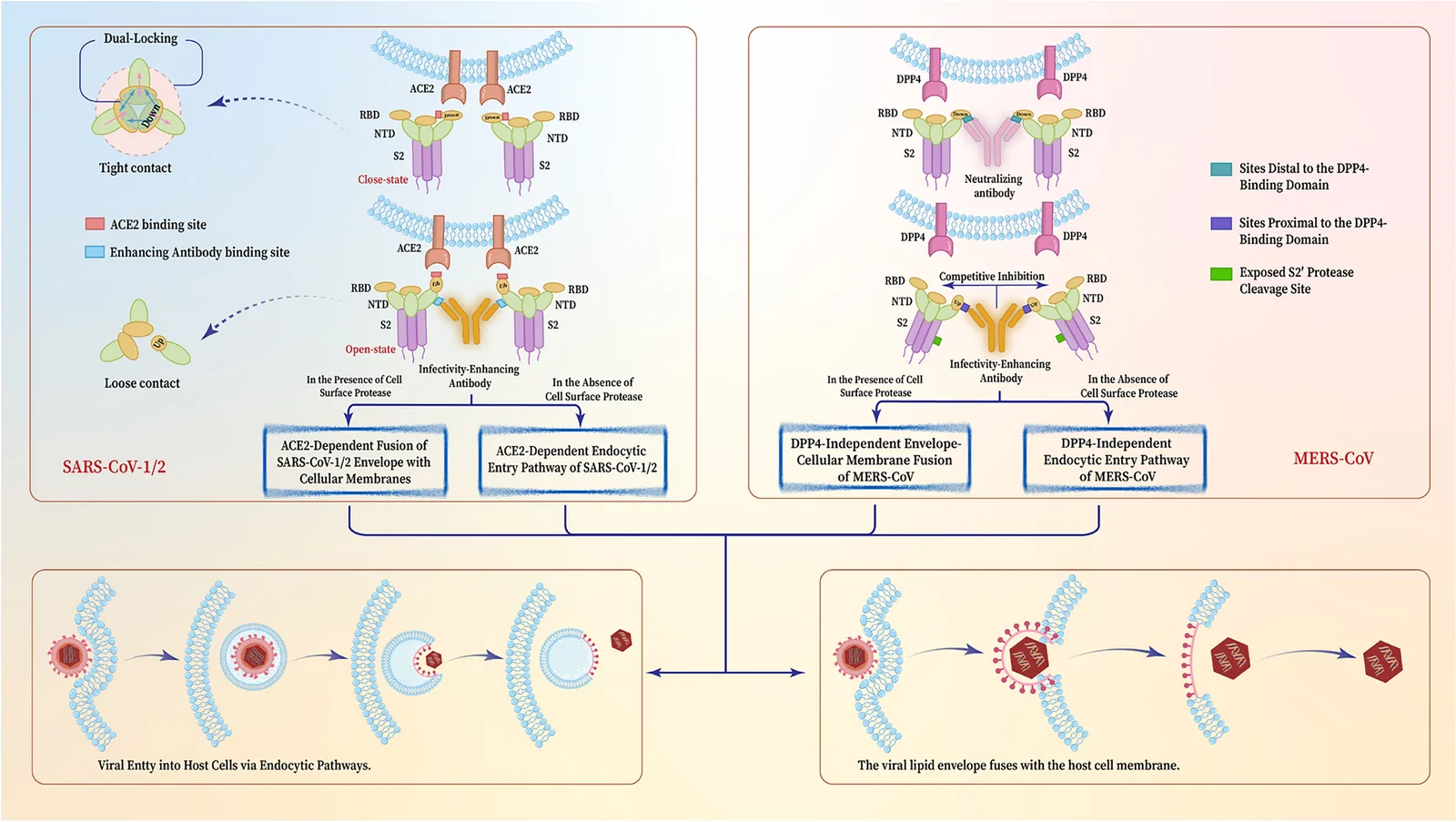
**Antibody-dependent conformational changes in viral proteins.** (i) Antibody-dependent conformational changes in SARS-CoV-1/2 spike protein: Infection-enhancing antibodies targeting the N-terminal domain (NTD) of the SARS-CoV-1/2 spike protein bind to a specific enhancing epitope within the NTD, thereby inducing an open conformation of the RBD. This conformational shift enhances the binding affinity between the spike protein and the host cell receptor ACE2, potentiating viral entry. (ii) Antibody-dependent conformational changes in MERS-CoV spike protein: The enhancing antibody Mersmab1 binds to the receptor-binding motif within the RBD of the MERS-CoV spike protein, stabilizing the RBD in an upright orientation. This stabilization triggers conformational rearrangements in the spike trimer, exposing the S2′ protease cleavage site. Notably, Mersmab1-dependent and DPP4-dependent MERS-CoV entry share a common downstream pathway, with the antibody effectively mimicking the native receptor to facilitate viral fusion and internalization.

An additional mechanism of ADE involves antibody-induced conformational changes in viral proteins that enhance virus-cell fusion. Guillon et al. [48] demonstrated in HIV infection that a gp120-specific monoclonal antibody recognizing the 16.1 subunit of the viral envelope glycoprotein could, at sub-neutralizing concentrations, bind and induce conformational changes in remaining subunits, thereby enhancing infection by promoting membrane fusion between the virus and target cells. Notably, this process was shown to be independent of the primary HIV receptor CD4 and was instead mediated through modulation of the interaction between gp120 and the viral co-receptor CCR5. Antibody-dependent conformational changes have also been reported during enhanced infection by coronaviruses. Wan et al. [7] showed that, akin to the native receptor DPP4, the enhancing antibody Mersmab1 binds to the same receptor-binding motif within the MERS-CoV RBD. This interaction stabilizes the RBD in an upright position and triggers conformational rearrangements in the S protein, exposing the S2′ protease cleavage site. Subsequent proteolytic cleavage of the S2 subunit induces dramatic conformational changes that initiate fusion between the viral envelope and host cell membrane, thereby enhancing viral entry and infection (Figure 3).

#### Antibody-dependent immune evasion

**Figure 4:**
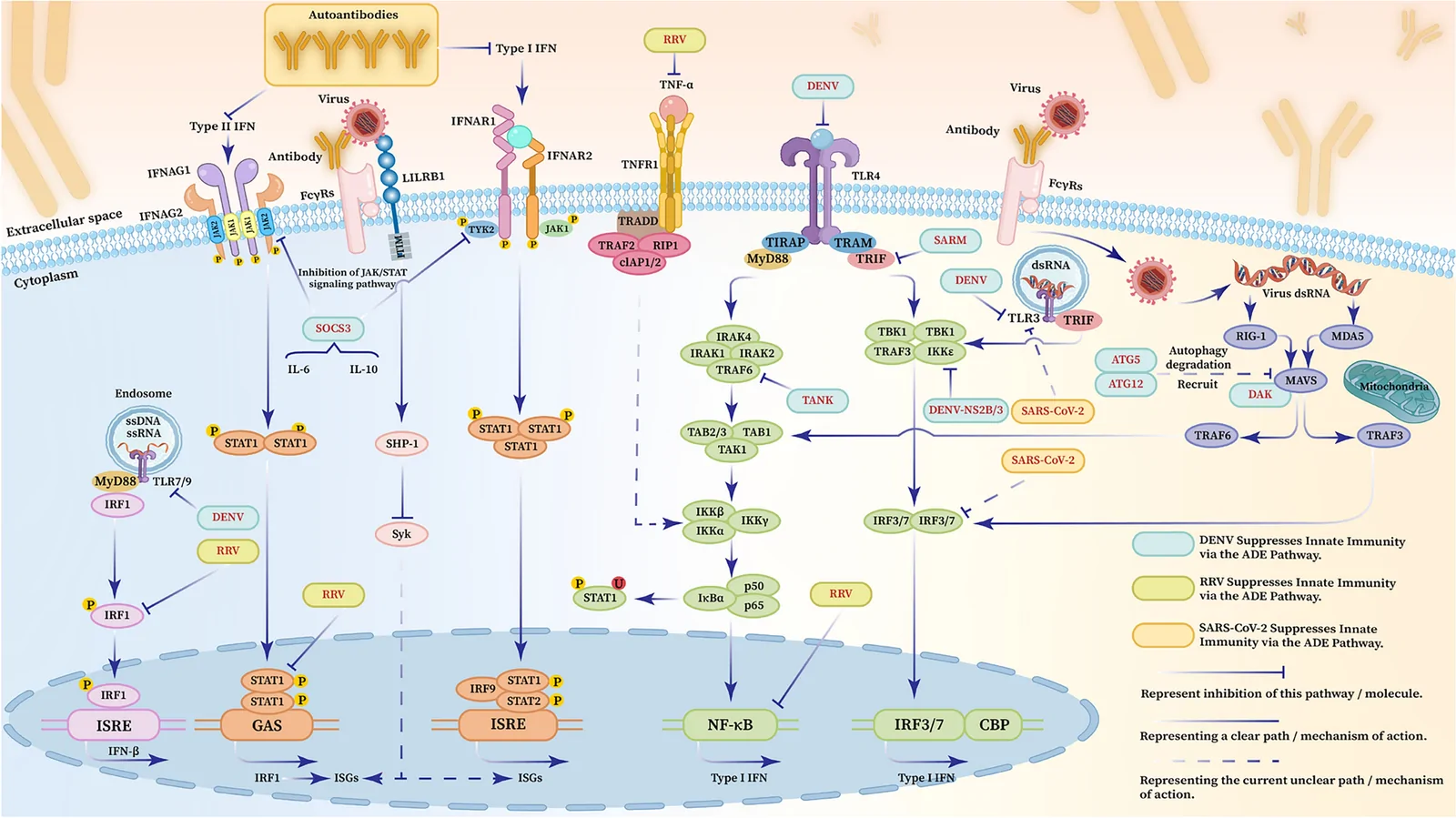
**Antibody-dependent immune evasion.** During ADE, immune-complex uptake can suppress antiviral innate signaling. In DENV models, ADE impairs TLR/RLR/JAK-STAT-associated pathways and reduces IFN-related responses. In RRV infection, ADE suppresses STAT1, NF-κB, and antiviral gene expression. In SARS-CoV-2 infection, anti-IFN autoantibodies provide a distinct example of antibody-dependent suppression of antiviral cytokine activity.

Upon viral infection, innate immune responses are initiated through TLR signaling, inflammasome activation (NLRP3/caspase‑1/IL‑1β), and cytosolic nucleic acid sensing (cGAS‑STING for DNA; RIG‑I/MDA‑5 for RNA), all of which drive type I IFN or inflammatory cytokine production [49–56].When virus-non-neutralizing antibody complexes are phagocytosed, the virus evades effective neutralization. As the complex dissociates from FcγRs, it can suppress the anticipated immune response, with type I IFN and pro-inflammatory cytokine production identified as primary targets of this suppression [57] (reviewed in Table 2 and Figure 4). Studies have shown that FcγRI and FcγRIIa synergistically promote DENV-antibody complex entry into THP-1 monocytes. Engagement of FcγR by DENV-antibody complexes not only downregulates TLR gene expression but also upregulates negative regulators of TLR signaling pathways, including SARM and TANK, leading to suppression of innate responses and increased viral production [9]. Furthermore, following entry into human monocytes, DENV-antibody complexes activate negative regulators dihydroxyacetone kinase (DAK) and the ATG5-ATG12 complex, which disrupt RIG-I/MDA-5 signaling cascades and impair type I IFN production, thereby suppressing interferon-mediated antiviral responses [1].

**Table 2:** **Mechanisms of viral suppression of innate immunity via ADE pathways**

Virus	Mechanisms of innate immune suppression	References
RRV	Inhibition of STAT1 activation thereby suppresses the transcriptional activation of IRF-1, IFN-β, and nitric oxide synthase 2 (NOS2). Concurrently, abolition of interferon-stimulated gene factor 3 (ISGF3) and NF-κB activity inhibits the expression of IP-10 and TNF-α	[62]
	Inhibiting NF-κB and IRF1, thereby suppressing NO activity and TNF-α expression	[3]
DENV	The ADE infection route of DENV elicits Th2-type immune response, upregulating the production of IL-10 and IL-6. This, in turn, leads to the overexpression of SOCS3, which subsequently suppresses the JAK-STAT signaling pathway and the production of IFN-γ	[1, 57]
	DENV, through co-engagement of FcγR and LILRB1 (an immunoreceptor with tyrosine-based inhibitory motifs), induces the dephosphorylation of SHP-1-a key regulator of FcγR signaling—thereby suppressing the type I ISGs response	[59]
	The engagement of DENV-antibody complexes with FcγR not only downregulates the expression of TLR genes (TLR3/4/7) but also activates the negative regulators of TLR signaling, SARM and TANK, which inhibit TRIF and TRAF-6, respectively	[9]
	Upon entry into human monocytes, DENV-antibody complexes activate negative regulators, including DAK and the ATG5-ATG12 complex. This disrupts the RIG-I/MDA5 signaling cascade and abrogates type I interferon production, leading to the suppression of interferon-mediated antiviral responses	[1, 63]
	The DENV NS2B/3 protein inhibits the kinase activity of IKKε	[64]
SARS-CoV-2	Auto-reactive antibodies capable of directly neutralizing IFNs are present in the peripheral blood of some COVID-19 patients	[10]
	Exosomes from COVID-19 patients contain auto-reactive antibodies targeting a variety of self-antigens, including immune-related factors such as chemokines, cytokines, and surface proteins	[65]
	Some COVID-19 patients harbor loss-of-function variants in loci related to the TLR3- and IRF7-dependent IFN-I pathway	[66]

As previously described, ADE begins with the binding of virus-antibody complexes to Fc receptors, which subsequently triggers Fc receptor-mediated endocytosis or phagocytosis. Engagement of activating Fc receptors initiates downstream signaling cascades that can induce the expression of interferon-stimulated genes (ISGs) independently of type I interferon signaling [58]. ISGs possess potent antiviral activities. Consequently, for ADE to occur productively, viruses must evolve mechanisms to counteract these antiviral responses within target cells (Figure 4). For example, dengue virus ADE additionally relies on engagement of the co-receptor LILRB1 [59]. LILRB1 signaling suppresses pathways that induce ISG expression, thereby creating an intracellular environment permissive for viral replication [36, 60]. Furthermore, while FcγR and cytokine signaling typically drive macrophage polarization toward a pro-inflammatory (M1) phenotype characterized by elevated production of reactive nitrogen and oxygen intermediates that enhance pathogen-killing activity [61], RRV infection of macrophages via ADE has been shown to downregulate nitric oxide synthase 2 (NOS2) and other immune factors, including interferon regulatory factor 1 (IRF-1), interferon-inducible protein 10 (IP-10), and interferon-beta (IFN-β), thereby facilitating viral immune evasion [62].

### ADEI

**Figure 5:**
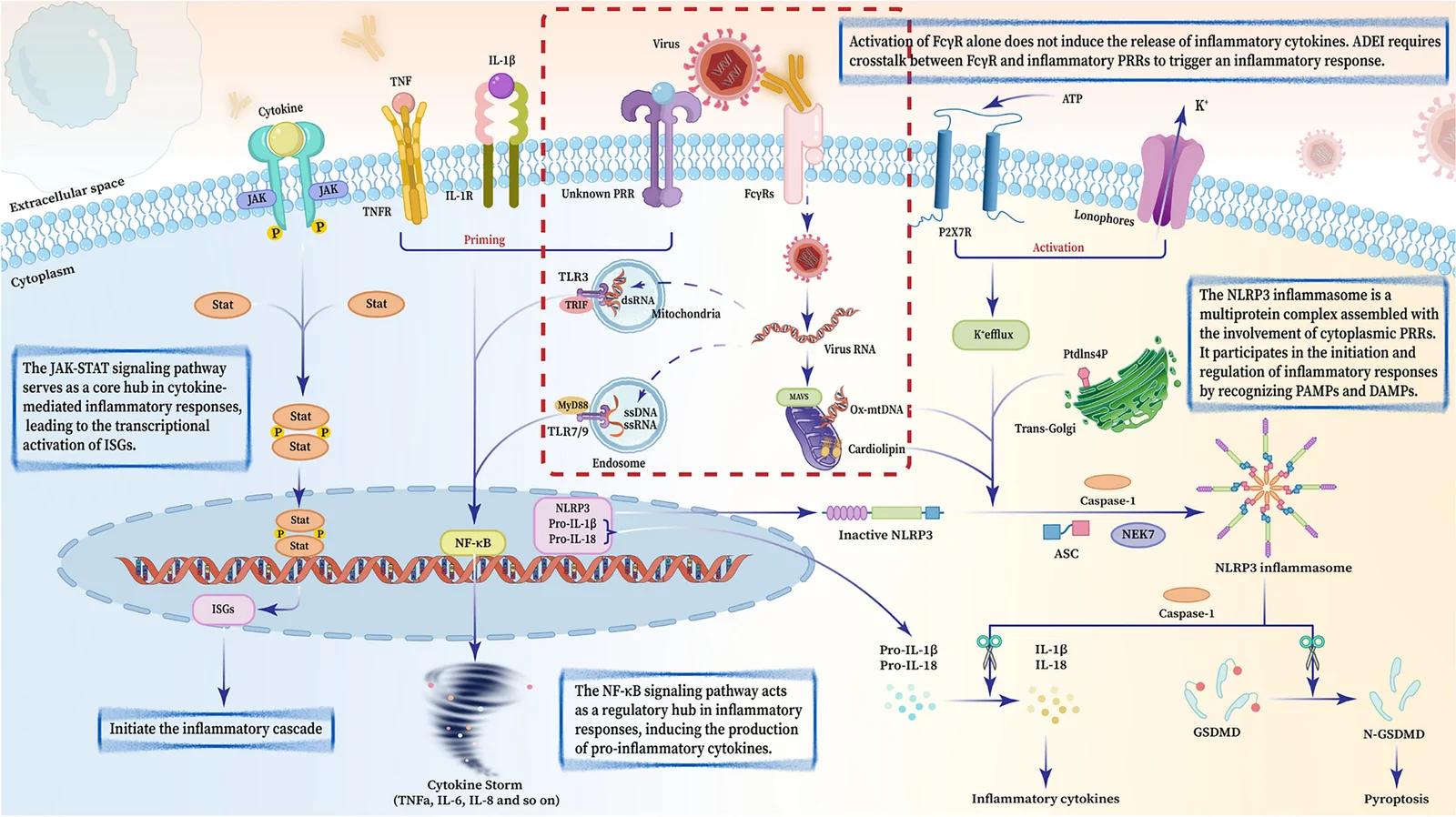
**Antibody-dependent enhancement of inflammation.** During ADEI, immune complexes amplify inflammatory responses through FcγR-PRR crosstalk, inflammasome signaling, and cytokine release. These pathways can promote NF-κB activation, NLRP3 inflammasome maturation, JAK-STAT-mediated cytokine amplification, pyroptosis, and tissue injury without necessarily increasing pathogen burden.

A key feature distinguishing FcγRs involvement in cytokine production is that FcγR themselves do not directly induce cytokine secretion; rather, they promote pro-inflammatory cytokine production through crosstalk with other co-receptors. Accordingly, one proposed mechanism for ADEI involves FcγR triggering of inflammatory responses via interplay with PRRs (Figure 5) [67]. This co-stimulation-dependent ADEI mechanism shares close parallels with FcγR-mediated cytokine induction observed during bacterial and protozoan infections. Studies have demonstrated that bacteria opsonized with serum IgG potently activate dendritic cells (DCs) and promote Th17 responses, upregulating IL-1β and IL-23 expression in an FcγRIIa-dependent manner. Notably, stimulation of FcγRIIa alone fails to induce cytokine production in DCs; instead, it selectively amplifies cytokine responses through synergistic interactions with TLR2, TLR4, or TLR5 [68]. During Leishmania infection, when amastigotes are coated with IgG, the resulting immune complexes engage FcγR on inflammatory macrophages, inducing robust IL-10 production while simultaneously reducing protective IL-12 levels, thereby exacerbating disease severity [69, 70]. Furthermore, antibodies may potentiate complement-driven inflammation, contributing to the exacerbated pathology observed during RSV [71] and influenza A virus (IAV) [72] infections. Of particular note, FcγR-mediated cytokine responses are governed by the delicate balance between activating and inhibitory FcγR. Studies show that stimulation of human DCs with IgG immune complexes simultaneously delivers inflammatory signals via activating receptor FcγRIIa and tolerogenic signals via inhibitory receptor FcγRIIb [73]. Dysregulation of this activating-inhibitory FcγR balance has been implicated in inflammation associated with bacterial infections [74]. On the other hand, TLR-FcγRIIa crosstalk in DCs promoting protective Th17 responses represents a mechanism for enhancing immunity, specifically facilitating pathogen clearance [68]. Thus, the defining characteristic of ADEI is the enhancement of pathological inflammation mediated by antibodies specific to a particular pathogen. However, unlike classical ADIPL, this process typically does not increase—and may even effectively reduce—pathogen load.

## ADE in animal coronaviruses and related porcine viruses

Coronaviruses are enveloped, positive-sense single-stranded RNA viruses belonging to the family *Coronaviridae* within the order *Nidovirales*. The family *Coronaviridae* is divided into two subfamilies: *Letovirinae* and *Orthocoronavirinae*. The latter is further classified into four genera: *Alphacoronavirus*, *Betacoronavirus*, *Gammacoronavirus*, and *Deltacoronavirus*. Prior to the outbreak of SARS in 2002, coronaviruses were not considered a major concern for human health; however, infections in animals—including pigs, cats, dogs, and poultry—have long been recognized as significant [75]. Historically, coronavirus-associated disease can be traced back to cases of feline infectious peritonitis (FIP) first documented in 1912 [75], and Feline infectious peritonitis virus (FIPV) was the first coronavirus unequivocally demonstrated to exhibit ADE [76].

### Feline coronavirus (FCoV)-mediated ADE

Based on amino acid sequence differences in the spike (S) protein, FCoV is classified into two serotypes: type I and type II FCoV. Each serotype can be further divided into two biotypes based on pathogenicity: FIPV and Feline Enteric Coronavirus (FECV). Using a specific pathogen‑free feline infection model, Takano et al. [77] investigated ADE induction by FIPV‑specific antibodies. Notably, ADE in FIPV exhibits stringent serotype‑dependence—enhancement occurs only when the pre‑existing antibodies and the challenging virus belong to the same serotype, whereas heterologous serotypes fail to provoke enhancement even in the presence of cross‑reactive antibodies. Their results demonstrated that passive immunization with type I FIPV‑specific antibodies significantly accelerated the progression of type I FIPV infection in cats, whereas no enhancement was observed in cats passively immunized with type II FIPV antibodies. Similar phenomena were observed in in vitro infection models. Studies showed that serum from cats that had been infected with type II FIPV strain 79–1146 and had seroconverted could enhance infection of primary feline alveolar macrophages and the human monocytic cell line U937 by the homologous FIPV 79–1146 strain; however, serum from cats infected with type I FIPV strains KU-2 or UCD-1 did not exhibit this enhancing effect [78]. These findings established a direct correlation between FIPV-induced ADE and serotype homology—in other words, antibodies possessing neutralizing activity also demonstrated potent ADE activity, confirming a close association between neutralizing and enhancing epitopes. However, neutralizing activity appears to be a sufficient but not necessary condition for ADE induction, as non-neutralizing monoclonal antibodies directed against the viral spike (S) and membrane (M) proteins have been observed to enhance FIPV replication in alveolar macrophages under certain circumstances [76]. A subsequent study characterized the relationship between ADE activity and immunoglobulin subclass among 19 neutralizing monoclonal antibodies specific to the FIPV S protein. The results indicated that most antibodies capable of both neutralizing and enhancing activities belonged to the IgG2a subclass [79]; however, exceptions existed within the enhancing epitope cluster A/A' (e.g., the IgG1 subclass monoclonal antibody 3G7 possessed both neutralizing and enhancing activities). This suggests that the ADE effect is not only related to the antibody subclass but is also closely associated with the specific epitope it binds to [80].

Current understanding suggests that FIPV arises from mutation of FECV, with the internal mutations conferring a tropism for monocytes/macrophages [81]. Specifically, FECV typically propagates within its natural host cells—mature intestinal epithelial cells—but upon adaptation for replication in monocytes/macrophages, viral virulence and biotype undergo alteration, resulting in the emergence of FIPV [82]. A monoclonal antibody against the FCoV spike protein (mAb 6–4–2) enhances FECV 79–1683 replication in U937 cells and primary feline monocytes, albeit with lower ADE activity than that observed for FIPV 79–1146. Moreover, FIPV ADE, but not FECV ADE, significantly upregulated inflammatory cytokines (TNF-α, IL-1β, IL-6) in primary monocytes [83]. Thus, while FECV infection of monocytes can be enhanced by antibodies, its replication efficiency remains lower than that of FIPV.

### CCV and its cross-reactive ADE in feline coronavirus infection

Although CCV primarily infects dogs and no direct evidence of CCV-specific ADE has been reported in its natural host, CCV is of particular relevance to the study of ADE in animal coronaviruses due to its close genetic and antigenic relationship with type II FCoV, its ability to infect cats subclinically, and its capacity to induce cross-reactive antibodies that can mediate ADE of FIPV infection [84–86]. Based on antigenic and genetic relationships with CCV, FCoV strains are classified into serotypes I and II. Antibodies against CCV can neutralize FCoV serotype II but not serotype I, indicating that serotype II FCoV strains are genetically more closely related to CCV. It has been proposed that serotype II FCoV strains arose through recombination events between serotype I FCoV and CCV [87, 88]. Although CCV is generally considered to associated with subclinical infection in cats, certain studies have demonstrated that some CCV strains can induce histological lesions in cats resembling those associated with enteric FCoV infection [89]. Furthermore, cats exposed to canine feces containing CCV may develop antibodies that cross-react with FCoV, and cats positive for CCV antibodies have been reported to experience ADE of FIPV infection [90].

### TGEV-mediated ADE

Evidence indicates antigenic cross-reactivity exists between FCoV and TGEV [91]. Furthermore, immunization of cats with recombinant vaccinia viruses expressing the spike glycoproteins of CCV and TGEV was demonstrated to enhance subsequent FCoV infection [92]. Given the high degree of antigenic relatedness between TGEV and FIPV, Olsen et al. [80] performed competitive ELISA (K-cELISA) cross-reactivity experiments using FIPV monoclonal antibodies and TGEV S protein-specific monoclonal antibodies whose epitopes had been previously mapped to defined regions of the TGEV S protein. The results revealed the presence of two major "enhancing epitope clusters" on the FIPV S protein, which were found to be homologous to the previously characterized sites A and E/F on the TGEV S protein. Moreover, monoclonal antibodies directed against TGEV sites A and E/F were also capable of inducing ADE of FIPV infection. This functional cross-reactivity provides further evidence for the close antigenic relationship between FIPV and TGEV.

### PEDV-mediated ADE

Evidence for PEDV-associated antibody-dependent enhancement (ADE) has been reported at both the monoclonal antibody and vaccine-immunization levels. Using phage display technology, Thavorasak et al. [93] identified an enhancing epitope in the spike (S) protein of PEDV. The monoclonal antibody mAbG3 was shown to recognize multiple discontinuous epitopes within the S1-0 subdomain of the PEDV S1 subunit and to enhance PEDV entry into susceptible Vero cells. Notably, because Vero cells do not express Fc receptors, this finding suggests that mAbG3-mediated enhancement may occur through an Fc receptor-independent mechanism rather than classical Fc receptor-mediated ADE.

In addition to monoclonal antibody-based evidence, vaccine-associated enhancement of PEDV disease has also been reported. Yu et al. [94] showed that immunization of piglets with a recombinant PEDV spike subunit vaccine, Bac-PEDV-S, induced detectable neutralizing antibodies before challenge; however, vaccinated piglets developed more severe diarrhea and shed higher levels of virus after homologous PEDV challenge than control animals. In that study, the Bac-PEDV-S group showed neutralizing antibody titers ranging from 40 to 160, with a mean FFN titer of approximately 110 in piglets, whereas the Bac-Ferritin control group did not induce positive neutralizing antibodies. Despite these neutralizing responses, the Bac-PEDV-S group exhibited more severe clinical signs and significantly higher fecal viral loads after challenge. The mechanism underlying this vaccine‑induced enhancement remains unresolved. One hypothesis is that the Bac‑PEDV‑S preparation, derived from crude insect cell lysates, contained both prefusion and postfusion forms of the spike protein. Since antibodies targeting the prefusion conformation are more potently neutralizing than those targeting the postfusion form [95, 96], non‑neutralizing antibodies directed against postfusion epitopes may have contributed to enhanced PEDV disease. However, this explanation remains speculative and requires further experimental confirmation.

### IBV-mediated ADE

Using a SPF embryonated chicken egg infection model, Reynolds et al. [97] evaluated ADE mediated by homologous antibodies against IBV. Briefly, IBV-immune antiserum (with an ELISA titer of 7463) was serially diluted twofold from 1:100 to 1:800, mixed with an equal volume of homologous IBV at a titer of 1 EID₅₀, and inoculated into SPF embryonated eggs. The results demonstrated that antiserum at a 1:100 dilution completely neutralized the virus, whereas the group treated with a 1:800 dilution of antiserum exhibited a statistically significant increase in the number of positive embryonated eggs compared to the group that received virus alone (*P* = 0.0457). This study provides the first preliminary evidence of IBV ADE using an embryonated chicken egg model with homologous antibodies. Given the substantial antigenic differences between vaccine and field strains [98], further investigation into ADE mediated by heterologous IBV antibodies is warranted.

### PRRSV-mediated ADE

Porcine reproductive and respiratory syndrome virus (PRRSV) causes severe reproductive failure and respiratory distress in pigs, resulting in substantial economic losses worldwide. ADE is a major obstacle to PRRSV vaccine development, as it can exacerbate disease severity. The phenomenon of ADE in PRRSV infection was first described in 1993, with a study demonstrating enhanced viral replication in fetuses inoculated with virus plus antibodies compared to those inoculated with virus alone [99]. Subsequent reports confirmed that sub-neutralizing concentrations of PRRSV-specific IgG elevated viremia and increased viral yield of heterologous strains [100, 101]. PRRSV exhibits a distinct pattern regarding strain homology compared to FIPV. In FIPV, ADE is strictly mediated by homologous antibodies [77, 78], whereas PRRSV displays the opposite pattern [102]. However, several studies reported that sub-neutralizing doses of PRRSV IgG did not induce ADE in vivo; instead, modified live-attenuated PRRSV vaccines conferred partial cross-protection against heterologous field strains [103–106]. This differential susceptibility may be associated with qualitative and quantitative differences in antigenic determinants [100]. Specifically, neutralizing epitopes are primarily located on the M, GP3, and GP5 proteins, whereas ADE-associated epitopes reside on the N and GP5 proteins. Variations in epitope sequences and glycosylation patterns among strains may skew vaccine-induced antibody responses toward either neutralization or enhancement [107]. Antibody-dependent immune evasion has been unequivocally demonstrated in PRRSV infection [108]. When PRRSV-antibody complexes enter cells via FcγR, they suppress transcriptional activation of IRF-1, IRF-3, and NF-κB, inhibiting antiviral genes TNF-α, IFN-α, and IFN-β [109, 110]. In porcine alveolar macrophages (PAMs), ADE pathway infection not only reduces synthesis of IFN-γ, IFN-λ1, IFN-λ3, and IFN-λ4 [111], but also upregulates IL-10 [112]. Three FcγR subtypes—FcγRI, FcγRIIb, and FcγRIII—participate in PRRSV ADE [112–115]. Antibody blocking assays revealed that the Sn1-6 ectodomain of porcine sialoadhesin (Sn) and the SRCR 5 domain of CD163 play central roles in FcγR-mediated ADE [116]. FcγRIIb-mediated ADE depends on its ITIM signaling, which recruits SHIP1 and suppresses the TBK1-IRF3 pathway, leading to downregulation of IFN-β transcription and enhanced viral replication [117]. FcγRI-mediated ADE antagonizes type I IFN secretion by interfering with the RIG-I/MDA-5 pathway, promoting PRRSV proliferation in PAMs [118]. Additionally, porcine FcεRI not only mediates ADE of PRRSV infection but also participates in antigen presentation and inflammatory regulation, reshaping macrophage immune responses to favor viral replication [119].

Beyond classical ADIPL, an atypical form of ADE involving FcγRI has been identified in PRRSV infection. Porcine FcγRI exists in at least six isoforms generated by alternative mRNA splicing [120, 121]. The membrane-bound isoform pCD64-T1 mediates classical ADE by internalizing PRRSV-antibody complexes, whereas the soluble isoform pCD64-T3 lacks the transmembrane domain and cannot mediate viral internalization. However, pCD64-T3 exhibits an "inflammation-enhancing effect": it is hypothesized to bind immune complexes and be captured by other PRRs, or act via paracrine signaling, reciprocally enhancing TLR pathway activity and upregulating pro-inflammatory cytokines including TNF-α, IL-1β, and IFN-β [122–124]. Given that this soluble FcγRI-mediated process does not increase pathogen load but promotes pathological inflammation, it can be categorized under the ADEI framework proposed in this review. In summary, PRRSV exemplifies both ADIPL—through FcγR-mediated immune evasion and enhanced viral entry—and ADEI—through soluble FcγRI-driven inflammatory amplification, underscoring the value of our unified outcome-based framework for dissecting these distinct pathogenic pathways.

To provide a systematic overview of the current knowledge base on ADE in the viruses mentioned above, we have summarized the key findings for each pathogen in Table 3. This table is structured to highlight, for each virus, the target cells, the receptor systems involved, and the specific ADE mechanism(s) documented—categorized according to our unified ADIPL/ADEI framework. Given the limited depth of mechanistic studies in most animal coronaviruses, we subsequently use SARS‑CoV‑2 as a case study (Sect. 4) to explore the full spectrum of potential ADE pathways that may also operate in livestock and poultry coronaviruses, thereby providing a valuable reference for future veterinary research.

**Table 3:** **Summary of ADE mechanisms reported in coronaviruses and related porcine viruses**

Virus	Host/target cells	Reported ADE mechanism(s)	Key observations	References
FIPV (type I & II)	Feline peritoneal macrophages	ADIPL (FcγR‑mediated); possible ADEI	ADE strictly homologous; both neutralizing and non-neutralizing mAbs can enhance; IgG2a subclass predominant for enhancement; enhanced inflammatory cytokine (TNF-α, IL-1β, IL-6) production	[76–80]
FECV	Feline monocytes/U937 cells	ADIPL (FcγR-mediated)	ADE activity lower than FIPV; does not induce inflammatory cytokine elevation in primary monocytes	[78]
CCV	Feline (cross-reactive)	ADIPL (FcγR-mediated)	Cross-reactive antibodies against CCV can enhance FIPV infection; serotype II FCoV shares genetic relatedness with CCV	[87–90]
TGEV	Feline (cross-reactive)	ADIPL (FcγR-mediated)	TGEV S protein shares enhancing epitope clusters (sites A and E/F) with FIPV; anti-TGEV mAbs induce ADE of FIPV infection	[80, 91, 92]
PEDV	Vero cells (no FcγR)/piglets	ADIPL (antibody-dependent conformational change)	mAbG3 binds discontinuous epitopes in S1-0 subdomain and enhances entry into FcγR-negative Vero cells; subunit vaccine-induced non-neutralizing antibodies (likely targeting postfusion S) enhance disease severity in piglets	[93, 94]
IBV	SPF embryonated chicken eggs	ADIPL (mechanism not fully characterized)	Homologous antiserum at sub-neutralizing dilution (1:800) significantly increased virus-positive egg rate; first in vitro evidence of IBV ADE	[97]
SARS-CoV-2 (case study)	Human monocytes, macrophages, B cells, epithelial cells	ADIPL (FcγR-mediated, C1q-mediated, conformational change); ADEI; immune evasion (ACA-mediated IFN neutralization)	Antibody-dependent conformational changes in NTD stabilize open RBD; afucosylated IgG drives inflammation; C1q-mediated ADE in FcγR-negative cells; autoantibodies against IFN-α/ω in severe patients	[10, 125–127]
PRRSV	Porcine alveolar macrophages (PAMs)	ADIPL (FcγR‑mediated immune evasion); ADEI (soluble FcγRI)	ADE involves Sn and CD163; ITIM‑SHIP1‑TBK1‑IRF3 pathway suppresses IFN‑β; RIG‑I/MDA‑5 pathway antagonized; soluble FcγRI (pCD64‑T3) amplifies inflammation without increasing pathogen load; heterologous ADE possible (unlike FIPV)	[116, 118, 124]

## Mechanisms of ADE in coronaviruses: a case study of SARS-CoV-2

The potential risk of ADE associated with SARS-CoV-2 infection, whereby pre-existing immunity from prior infection or vaccination may lead to exacerbated disease, has emerged as a public health concern of global significance [128, 129]. Immune complexes formed by viruses and antibodies (primarily non-neutralizing or cross-reactive antibodies) can bind to FcγR and undergo internalization, thereby enhancing viral entry [11, 18]. It has been generally assumed that FcγR are predominantly expressed on myeloid cells, which are not considered the primary targets of SARS-CoV-2 infection; consequently, the clinical significance of ADE in SARS-CoV-2 infection has remained a subject of debate. However, the detection of viral RNA in macrophages, neutrophils, plasma B cells, and T lymphocytes obtained from bronchoalveolar lavage (BAL) fluid [17] indicates that SARS-CoV-2 infection is not restricted to the respiratory tract [130], necessitating a reassessment of the clinical relevance of ADE in SARS-CoV-2 pathogenesis. Currently, five major mechanisms have been proposed to underlie SARS-CoV-2 ADE: (i) Fc receptor-mediated enhancement of SARS-CoV-2 infection; (ii) complement receptor-mediated enhancement of SARS-CoV-2 infection; (iii) antibody-dependent conformational changes in the SARS-CoV-2 spike protein facilitating enhanced internalization; (iv) SARS-CoV-2-specific ADEI; and (v) SARS-CoV-2 antibody-dependent immune evasion.

### Fc receptor-mediated enhancement of SARS-CoV-2 infection

FcγR-mediated ADE has been previously documented for SARS-CoV-1 and MERS-CoV infections [7, 131]. Studies on SARS-CoV-2 have demonstrated that human monoclonal antibodies (mAbs) targeting the spike protein can enhance viral infection of human B lymphoblastoid and chronic myeloid leukemia cell lines through FcγR-dependent pathways in vitro [132, 133]. One study employing pseudotyped virus, serial dilutions of mAbs, and various cell lines evaluated SARS-CoV-2 ADE and the potential involvement of specific FcγR genotypes. The results showed that the neutralizing mAb MW05 enhanced SARS-CoV-2 pseudovirus infection of Raji cells but had no effect on infection of THP-1 or K562 cells. Characterization of FcγR expression profiles by flow cytometry revealed that Raji cells—which exhibited ADE activity with MW05—expressed relatively high levels of FcγRIIB exclusively; THP-1 cells expressed high levels of FcγRIA and FcγRIIA; while K562 cells expressed only high levels of FcγRIIA. These findings suggest that FcγRIIB is the primary Fcγ receptor mediating MW05-enhanced SARS-CoV-2 infection [134]. Using COVID-19 convalescent plasma and BHK cells stably expressing human FcγRs and hACE2, [135] assessed ADE of SARS-CoV-2 infection. Their results indicated that FcγRIIA and FcγRIIIA could mediate mild ADE of SARS-CoV-2 infection; however, this effect required the participation of FCER1G, the gamma chain subunit of the IgE receptor. Notably, discrepant conclusions regarding the role of FcγRIIA in mediating ADE have been reported by different research groups. At least two factors may account for these apparent discrepancies. First, not all cell types expressing FcγRIIA are capable of supporting FcR-dependent viral entry; host–pathogen interactions at the cell surface may expand target cell susceptibility only when coupled with hACE2 [136]. Second, different FcγRs exhibit varying affinities for antibody subclasses based on their amino acid sequences, with preferential binding to particular IgG subtypes. This differential binding capacity depends on the antibody type, the FcγR genotype, and its expression level on the cell. Consequently, when analyzing FcR-mediated antibody functions, consideration must be given to host FcR polymorphisms [46].

Given that ACE2 is essential for SARS-CoV-2 entry, it is intriguing to explore antibody-mediated enhancement via a dual-receptor mechanism involving both FcγRs and ACE2 [136]. Multiple reports have demonstrated that certain plasma samples from COVID-19 patients enhance SARS-CoV-2 infection exclusively in cells expressing both Fc receptors and ACE2 [137, 138]. Additional evidence supports a critical role for ACE2 in FcγR-dependent ADE of SARS-CoV-2 infection. Specifically, in cells expressing FcγRs but lacking ACE2, or in cells co-expressing ACE2 and FcγRs in the presence of high concentrations of neutralizing antibodies, viruses can be bridged to FcγRs via antibodies but fail to engage ACE2. Lacking the ACE2-induced conformational change in the S protein required for subsequent entry steps, the virus ultimately traffics to and is degraded in lysosomes. In contrast, in cells co-expressing ACE2 and FcγRs, subneutralizing antibody concentrations promote viral attachment to the cell via Fc-FcγR interaction, while allowing unneutralized S proteins to bind ACE2 and initiate the entry program. Thus, in the presence of sub-neutralizing antibodies, FcγRs function as attachment factors that enhance virus receptor-mediated invasion, with ACE2 potentially serving as a secondary receptor participating in antibody- and FcγRs-mediated ADE of SARS-CoV-2 infection [139].

### Complement receptor-mediated enhancement of SARS-CoV-2 infection

It has been suggested that the clinical relevance of FcγR-mediated ADE may be limited, as FcγRs are not expressed on the primary target cells of SARS-CoV-2 infection [140]. Okuya et al. [127] demonstrated that COVID-19 convalescent serum supplemented with exogenous C1q (at a concentration of 50 μg/mL) could enhance SARS-CoV-2 infection of Vero E6 cells, which lack Fcγ receptors—a phenomenon attributed to C1q-mediated ADE. This mechanism may be particularly relevant to respiratory epithelial cells, the primary targets of SARS-CoV-2 infection, given that C1q is abundantly present in plasma and C1q receptors are widely expressed on various cell types [141]. Considering that among animal coronaviruses with documented ADE, only FIPV targets peritoneal macrophages expressing FcγR, greater attention should be directed toward C1q-mediated ADE in the context of animal coronavirus infections.

### Antibody-dependent conformational changes in the SARS-CoV-2 spike protein facilitating enhanced internalization

During its functional cycle, the spike protein undergoes several distinct conformational transitions [142–145]. Coronavirus S protein‑mediated entry involves S1/S2 proteolytic cleavage, RBD‑receptor complex formation, membrane fusion, and release of the viral genome into the cytoplasm [95]. SARS-CoV-2 enters cells through binding of its RBD within the S1 subunit to ACE2, followed by proteolytic activation. The native spike exists as a homotrimer and adopts two principal conformations. In the closed conformation, all three RBDs are in a “down” position, rendering the receptor-binding sites inaccessible. In the open conformation, at least one RBD adopts an “up” state, exposing the receptor-binding interface and enabling its interaction with ACE2, thereby facilitating viral attachment and entry. Studies have shown that in SARS-CoV, the RBD predominantly adopts a standing (up) conformation [146, 147], whereas in SARS-CoV-2, the RBD predominantly adopts a lying (down) conformation [95, 148]. Certain antibodies targeting the NTD of the SARS-CoV-2 spike protein, identified in COVID-19 patients, have been demonstrated to induce the open conformation of the RBD. This conformational shift enhances the binding affinity between the spike protein and ACE2, thereby augmenting viral infectivity (Figure 3) [125]. Further investigation revealed that these enhancing antibodies anchor the NTD within lipid raft microdomains, stabilizing the interaction between the spike trimer and the host cell membrane. This stabilization mechanism may promote conformational changes in the spike, consequently exposing the RBD. Notably, the NTD also serves as a target for neutralizing antibodies. In individuals vaccinated with spike protein vaccines (including mRNA or viral vector vaccines) derived from the original Wuhan strain, the balance between neutralizing and enhancing antibodies favors neutralization [149]. However, against the Delta variant, neutralizing antibody affinity is substantially reduced, while enhancing antibody affinity is markedly increased [149].

Given that the open conformation of the SARS-CoV-2 spike exposes the receptor-binding domain and enables ACE2 engagement, researchers have explored strategies to stabilize the spike in a closed, receptor-inaccessible conformation to restrict viral attachment and entry. For instance, Schoof et al. [150] engineered a multivalent single-chain antibody by linking two distinct RBD-specific monoclonal antibodies via a GS linker, which effectively stabilized the spike in the closed conformation and abrogated SARS-CoV-2 infectivity. Another study investigated N-acetylcysteine (NAC) as a means to perturb the critical binding affinity between the spike protein and ACE2. Results demonstrated that NAC binding reduced the spike-ACE2 binding affinity by three-fold and decreased the free energy of release (ΔG) by 5 kcal mol⁻^1^ [151]. This work represents a valuable exploration of "artificial perturbation of viral protein conformation to attenuate virus-host interactions," offering novel insights into the development of adjunctive therapeutics against COVID-19.

### SARS-CoV-2-specific ADEI

Research on the roles of antibodies and FcγR during viral infection has primarily focused on virus neutralization, ADCC, ADCP, and ADE of infection. In contrast, FcγR-mediated cytokine responses in the context of viral infection have received comparatively less attention. Studies have demonstrated that monocytes, dendritic cells, and macrophages exposed to serum-opsonized dengue virus upregulate TNF-α and IL-6 production in an FcγRIIa-dependent manner [152]. This response may arise from crosstalk between FcγRIIa and TLR3 or TLR7/8, which recognize viral double-stranded and single-stranded RNA, respectively [153]. Although the precise mechanisms underlying FcγR crosstalk with other receptors in mediating pathogen-specific cytokine responses have not been fully elucidated in the context of SARS-CoV-2 infection, the enhancement of pathological inflammatory responses mediated by SARS-CoV-2 antibodies has been well documented in both in vitro and in vivo studies. Research has shown that SARS-CoV-2 can infect monocytes via binding of specific serum IgG to FcγRIII, supporting the existence of ADE. However, SARS-CoV-2 ADE differs from classical ADE in two key aspects. First, infection of monocytes does not primarily serve viral replication. Second, infected monocytes undergo pyroptosis, forming inflammasomes that serve as major sources of inflammatory cytokines; disease severity in patients correlates positively with the extent of pyroptosis and levels of inflammatory factors [154]. Further investigation revealed that afucosylated anti-spike IgG antibodies drive the production of inflammatory cytokines and immune cell infiltration into the lungs of severe COVID-19 patients [126].

Two clinical case reports provide compelling support for antibody-induced enhancement of inflammatory responses. One case described a 75-year-old patient who developed cytokine release syndrome (CRS) within four hours of pre-infusion with anti-SARS-CoV-2 antibodies (casirivimab/imdevimab). Elevated serum levels of IL-6, TNF-α, IL-8, and IL-10 triggered immune activation, ultimately leading to progressive acute respiratory distress syndrome [25]. Another case report documented acute exacerbation of COVID-19 pneumonia severity following casirivimab/imdevimab infusion, consistent with ADE. Prior to infusion, the patient exhibited mild COVID-19 symptoms with normal oxygen saturation; post-infusion, the patient developed acute hypoxic respiratory failure [26]. These observations suggest that inflammatory responses in the airway epithelium play a critical role in SARS-CoV-2 pathogenesis. Antibodies may exacerbate lung pathology by activating macrophages, even if they do not necessarily increase viral load and dissemination as seen in classical ADE [155]. Additionally, Adeniji et al. [156] found that antibodies from severe COVID-19 patients induced high levels of antibody-dependent complement deposition (ADCD), which may contribute to the elevated inflammation associated with severe disease. Notably, similar immunological features have been reported in feline infectious peritonitis (FIP) cases, where production of the pro-inflammatory cytokine TNF-α contributes to disease exacerbation. Increased TNF-α production correlates with viral replication and ADE in FCoV-infected macrophages. Elevated TNF-α levels can act on macrophages to promote FCoV receptor expression and are responsible for inducing apoptosis of CD8+ T cells [157, 158].

In summary, ADEI may lead to more severe disease, triggering cytokine storms and further tissue damage [26]. Conversely, excessive suppression of immune factors may facilitate antibody-dependent immune evasion. Therefore, maintaining an appropriately balanced immune response during infection may represent an effective strategy for avoiding coronavirus ADE.

### SARS-CoV-2 antibody-dependent immune evasion

The mechanisms by which SARS-CoV-2 enhances infection through antibody-dependent immune evasion differ in certain aspects from those described in Sect. 2.1.4. Specifically, in addition to suppressing the expression and release of IFN-I, ISGs, and other immune factors via signaling pathways, SARS-CoV-2 enhancing antibodies may also act through a more direct mechanism: certain autoantibodies can directly neutralize IFNs. Analysis of peripheral blood from 987 patients with severe COVID-19 revealed that at least 101 individuals (10.2%) possessed autoantibodies against IFN-ω (13 patients), one or more of the 13 IFN-α subtypes (36 patients), or both (52 patients) at the time of severe disease; a minority also had autoantibodies against other type I IFNs. These antibodies demonstrated the capacity to neutralize IFNs and abrogate their ability to block SARS-CoV-2 infection in vitro. In contrast, such autoantibodies were not detected in 663 individuals with asymptomatic or mild COVID-19. This study suggests that these IFN autoantibodies may be implicated in the development of life-threatening COVID-19 in at least 2.6% of women and 12.5% of men [10]. Further sequencing of samples from 659 severe and 534 mild/asymptomatic patients revealed an enrichment of rare loss-of-function (LOF) variants in IFN-I-related gene loci dependent on TLR3 and IRF7—genes previously implicated in influenza virus immunity. Detailed analysis identified LOF variants in 23 patients (3.5%). TLR3- and IRF7-dependent IFN-I immunity constitutes a critical component of innate antiviral defense; mutations in this pathway may thus provide an intrinsic mechanism underlying SARS-CoV-2 enhancement through antibody-dependent immune evasion [66]. Another study analyzed and characterized 2770 exosomes from 194 COVID-19 patients, finding that many possessed auto-reactive antibodies against a variety of self-antigens, including immune-related factors such as chemokines, cytokines, and surface proteins. Notably, autoantibodies against the IFN-I pathway were particularly enriched in severe cases. The presence of these autoantibodies correlated significantly with virological and immunological parameters such as viral load, antibody titers, and immune cell counts. Subsequent validation in a mouse model demonstrated that autoreactive antibodies altered peripheral immune cell composition, suppressed antiviral immunoregulatory pathways, and exacerbated infection severity and mortality [65].

## Concluding remarks

The primary function of virus-specific antibodies is to neutralize viral infection and thereby contribute to host antiviral defense. According to the multi-hit kinetic model of virus neutralization, effective neutralization requires sufficient antibody occupancy on each virion to exceed the stoichiometric threshold for neutralization [159]. However, when antibodies are present at sub-neutralizing concentrations or lack neutralizing activity, they may fail to block infection and instead facilitate viral entry into primary target cells or otherwise non-permissive secondary target cells, thereby expanding viral tropism [160]. In addition, ADE may modulate intracellular signaling pathways and suppress antiviral immune responses. These mechanisms, acting individually or in combination, can contribute to enhanced viral infection and disease progression.

ADE has been documented for numerous viruses, including DENV, HIV, EBOV, RSV, and SARS-CoV-1 [31]. PRRSV, although not a coronavirus, provides a valuable veterinary example showing that antibody-mediated entry, immune evasion, and inflammatory amplification can coexist during ADE [116, 118, 124]. In contrast to DENV, for which the association between ADE and severe disease is well established, the clinical relevance of ADE in coronavirus infections remains incompletely understood [65]. For example, antibodies targeting the RBD and NTD of the SARS-CoV-2 spike protein can mediate both neutralization and in vitro enhancement of infection, yet these antibodies showed antiviral effects in mouse and non-human primate models [161]. Similarly, ADE has been observed in experimentally vaccinated cats [162, 163], and in some feline coronavirus vaccine trials [164, 165], whereas field studies of vaccinated cats have not demonstrated clear evidence of ADE [166]. One possible explanation is that post-vaccination serum contains a complex mixture of neutralizing, non-neutralizing, and potentially enhancing antibodies; therefore, whether infection is blocked or enhanced may depend on the quantitative and functional balance among these antibody populations [167].

Although pre-existing immunity induced by coronavirus vaccines may carry a potential ADE risk, vaccines remain among the most effective tools for preventing and controlling animal infectious diseases. The key challenge is therefore not to avoid antibody induction, but to optimize antibody quality, epitope specificity, and immune polarization. Several strategies may help reduce ADE risk. For SARS-CoV-2, neutralizing epitopes are concentrated within the receptor-binding motif of the RBD, whereas non-neutralizing epitopes outside this region may be shielded by glycosylation to reduce undesirable antibody responses [168]. For MERS-CoV, S1-based vaccines have been reported to induce better protective immunity with fewer ADE concerns than full-length S protein vaccines [169]. Similarly, the FIPV S2 subunit lacks identified ADE epitopes, making S2-targeted antigen design a potential strategy [170], and the S2-derived FP-HR2 peptide can elicit both Th1 and Th2 immune responses in mice [171]. Beyond antigen selection, immune response type may also influence ADE and immunopathology: Th2-biased responses have been associated with enhanced disease in some vaccine settings [169], whereas Th1-skewed immunity may provide protection while reducing immunopathological injury [172, 173].

Most coronaviruses, except FIPV, primarily infect epithelial cells rather than FcγR-rich macrophages. Therefore, when evaluating vaccine-induced ADE risk in veterinary coronaviruses, particular attention should be paid not only to FcγR-mediated pathways but also to complement receptor-mediated ADE and antibody-induced conformational changes, both of which may facilitate infection of non-myeloid cells lacking FcγR. ADEI should also be considered, as it shares mechanistic parallels with vaccine-associated enhanced respiratory disease (VAERD), first described after formalin-inactivated RSV vaccination [16]. Overall, ADE in animal coronaviruses remains incompletely defined, and future studies should distinguish ADIPL from ADEI, evaluate antibody function in host-relevant cell models, and determine whether experimentally observed ADE has true relevance under field conditions.

## Data Availability

No datasets were generated or analysed during the current study.
